# Occupational stress and burnout among teachers in China: latent profile and network analyses

**DOI:** 10.3389/fpsyg.2026.1862159

**Published:** 2026-07-29

**Authors:** Jinhua Cai, Qiao Zhang, Yifan Wang, Yujia Meng, Haijun Duan

**Affiliations:** 1 School of Teacher Development, Shaanxi Normal University, Xi’an, China; 2 Xi’an No.1 Experimental Kindergarten, Xi’an, China; 3 School of Psychology, Shaanxi Normal University, Xi’an, China; 4 Key Laboratory of Modern Teaching Technology, Shaanxi Normal University, Xi’an, China; 5 Faculty of Education, Shaanxi Normal University, Xi’an, China

**Keywords:** latent profile analysis, network analysis, occupational burnout, teacher stress, teacher well-being

## Abstract

**Introdution:**

Severe occupational stress and burnout are widespread among teachers in primary and secondary schools in urbanizing China. While studies have examined these phenomena, few studies have used an individual-centered perspective meanwhile integrated Latent Profile Analysis (LPA) and Network Analysis (NA) to investigate the heterogeneity and underlying connections between stress and occupational burnout.

**Methods:**

This study aimed to address these gaps by examining co-occurrence patterns and core symptoms of teacher stress and burnout in a sample of 2,769 teachers using the Wilson Stress Profile and the Maslach Burnout Inventory.

**Results:**

The LPA revealed two distinct latent profiles: low stress-burnout and high stress-burnout. NA showed heterogeneity in the stressburnout network, with physical symptoms central in the low stress-burnout group and emotional exhaustion in the high stress-burnout group.

**Discussion:**

The networks indicated strong interconnections, suggesting a dynamic link between stress and burnout. These findings highlight the varied experiences of teacher stress and burnout, informing targeted interventions to improve teacher well-being.

## 1 Introduction

With the increasing intensity of social competition in the work environment, teachers in China face an escalating workload and longer working hours (Teng et al., 2024), leading to heightened stress and occupational burnout (Kijima et al., 2020). Persistent and severe stress and occupational burnout among teachers are widespread within the educational sector (Cheng et al., 2023), significantly detriment not only teachers’ work performance (Perlito et al., 2021) and well-being (Zhong et al., 2009), but also students’ academic performance (Madigan and Kim, 2021) and future development (Herman et al., 2018).

Stress encompasses the negative subjective experiences, as well as the psychological and physiological responses, triggered by various adverse stimuli in the work environment (Austin et al., 2005). Luh et al. (1991) identified multiple sources of teacher stress, including student behavior, interpersonal and organizational relationships, time management, and psychological and physical stress-related factors, highlighting the complexity of stressors in the teaching profession and the need for targeted interventions. Previous research predominantly explored the connection between stress and occupational burnout through frameworks such as occupational demands, control, social support, and available resources, revealing a strong link between stress and occupational burnout (Herman et al., 2018).

Occupational burnout is a multidimensional psychological syndrome characterized by chronic emotional and physical exhaustion resulting from prolonged stress (Maslach and Jackson, 1981). It encompasses three dimensions: emotional exhaustion, depersonalization, and diminished personal efficacy (Maslach and Jackson, 1981; Park and Shin, 2020). Emotional exhaustion reflects depleted emotional and physical resources, depersonalization refers to disengagement from work and interpersonal relationships, and reduced personal accomplishment involves a decline in perceived competence and achievement (Zhao and Jiang, 2022; Makara-Studzińska et al., 2019). These dimensions are interrelated yet distinct, suggesting that burnout may be better understood as a system of interacting components rather than a single uniform construct (Xie et al., 2022).

Studies have shown that stress and occupational burnout are related (Herman et al., 2018; Queirós et al., 2020). As stress intensifies, occupational burnout similarly increases (Maddock, 2024; Queirós et al., 2020; Zhao and Jiang, 2022). The co-occurrence of stress and occupational burnout is commonly observed in various professions, including but not limited to researchers (Gao et al., 2023), journal editors, doctors (McManus et al., 2002), healthcare workers (Zhou et al., 2020), police officers (Queirós et al., 2020). Furthermore, studies have utilized the same intervention strategies to mitigate both stress and job burnout (Durden et al., 2023; Ghannam et al., 2020; Innstrand et al., 2004; Suyi et al., 2017), suggesting that these stress and occupational burnout share common psychological and physiological mechanisms. Thus, the concurrent presence of stress and occupational burnout is a widely observed pattern.

Key theoretical models in this domain, like the Occupational Demands-Control Model and the Occupational Demands-Resources Model (Bakker and Demerouti, 2007), proposed that individuals facing stress must deplete internal resources to effectively resolve physical and psychological efforts. When internal psychological resources are limited and demanded, stress levels may escalate, eventually depleting both physical and mental resources, thereby leading to occupational burnout (Bakker and Demerouti, 2007). Some studies suggested that stress serves as a precursor to occupational burnout (Guthier et al., 2020). Emotional exhaustion, a core component of burnout, is particularly sensitive to work-related stress, reflecting its direct impact. Maslach and Jackson (1981) pointed out that emotional exhaustion sets off a cascade of adverse psychological responses, leading individuals to erect psychological barriers against stressors, which ultimately results in depersonalization (Maslach and Jackson, 1981). This, in turn, undermines work performance and self-esteem, thereby exacerbating the risk of occupational burnout and a decline in perceived personal achievement. Consequently, occupational stress is correlated with job burnout. Despite their strong relationship, stress and occupational burnout exhibit conceptual, dimensional, and symptomatic differences. Maslach contends that stress is not an inevitable precursor to occupational burnout, but rather, it is the prolonged exposure to stress without relief that triggers occupational burnout (Maslach and Leiter, 2016).

While extensive research has been conducted on stress and occupational burnout, a notable remains regarding a unified framework that integrates both teacher stress and burnout and systematically explores their co-occurrence patterns. Current research predominantly addresses these phenomena in isolation, or focuses on isolated intervention strategies, leaving a limited understanding of how stress and burnout coexist in teachers and how these experiences vary across subgroups. Moreover, the co-occurrence patterns are rarely examined through methodologies that allow for the exploration of internal relational networks within subgroups (Akbari et al., 2024; Peng and Liao, 2023; Wang et al., 2023; Xue et al., 2024; Zhou Y. et al., 2024). Thus, this study aims to address these limitations by integrating Latent Profile Analysis (LPA) and Network Analysis (NA) to identify severity-based subgroups of teachers and explore the network structure of stress and occupational burnout within these subgroups.

As a person-centered approach, LPA can identify subgroups of teachers with different levels of stress and occupational burnout. However, it does not provide information about how stress and burnout dimensions are associated within each subgroup. Network analysis complements this limitation by examining the conditional associations among stress and burnout dimensions and identifying potentially influential nodes within the network. Therefore, integrating LPA and network analysis enables the investigation of both subgroup differences and within-group relational patterns, providing a more comprehensive understanding of the co-occurrence of teacher stress and occupational burnout (Fried and Cramer, 2017). Given their unique role and responsibilities in the education system, teachers face challenges such as high emotional labor, long working hours, and complex interpersonal dynamics, which contribute to stress and occupational burnout. This study addresses two central theoretical questions: (1) What subgroups characterized by different levels of stress and occupational burnout can be identified among teachers in China? and (2) How do the network structures of stress and burnout differ across these profiles, and which symptoms emerge as central or bridge nodes within these networks? By identifying these patterns, this study aims to provide preliminary evidence for future intervention research and to enhance understanding of how stress and occupational burnout are associated among teachers.

## 2 Materials and methods

### 2.1 Procedure

Subjects were recruited for the Project of Compulsory Education Quality Monitoring. Data were collected using an online survey platform, which is the most widely used data collection tool in mainland China. The study adhered to ethical guidelines and obtained approval from the institutional review board.

### 2.2 Participants

Participants included primary and secondary school teachers from Shaanxi Province, China. A total of 2,769 teachers completed the survey. Descriptive statistics for the sample were provided in Table 1. Participants reported an average teaching experience of 11.46 years (range: 0–48 years). The majority of the teachers identified as female (66.9%).

**TABLE 1:** Sample demographics.

Descriptor	*n*	%
Gender		
Female	1852	66.9
Male	917	33.1
Age		
20–29	605	21.8
30–39	879	31.7
40–49	976	35.2
> 50	309	11.2
Occupational tenure		
< 5 years	732	26.4
5∼10 years	396	14.3
11∼20 years	624	22.5
≥ 21 years	1017	36.7
Educational background		
Secondary Education	343	12.4
Bachelor	2319	83.7
Master or above	107	3.9

### 2.3 Measures

The questionnaire used in this study consisted of a section for sociodemographic information and two well-established measurement tools. The details of these instruments are outlined below:

The Wilson Stress Profile for Teachers (WSPT, Luh et al., 1991) was used to assess teacher stress. The original English version includes 36 items across nine subscales: (1) Student Behavior (SB), (2) Employee-Administrator Relationships (EAR), (3) Teacher-and-Teacher Relations (TTR), (4) Parent-Teacher Relations (PTR), (5) Time Management (TM), (6) Intrapersonal Conflicts (IC), (7) Physical Symptoms of Stress (PS), (8) Psychological/Emotional Symptoms of Stress (PES), and (9) Stress Management Techniques (SM). Each subscale contains four items, and responses are rated on a 5-point Likert scale from 1 (never) to 5 (very often). The research team developed a Chinese version following a standard translation–back-translation procedure. Two bilingual experts in educational psychology independently translated the original English items. The Cronbach’s alpha for the subscales in this study was 0.65 (SB), 0.87 (EAR), 0.83 (TTR), 0.89 (PTR), 0.81 (TM), 0.52 (IC), 0.87 (PS), 0.90 (PES), and 0.67 (SM). Confirmatory factor analysis (CFA) using the robust maximum likelihood estimator (MLR) in lavaan (Rosseel, 2012). Given the ordinal nature of the Likert-scale items and potential non-normality, MLR was employed to provide scaled test statistics and robust standard errors. The initial 36-item model produced a Heywood case (negative residual variance for PTR4), indicating empirical underidentification. Following psychometric recommendations (Chen et al., 2001), we removed PTR4 and re-estimated the model with 35 items. The nine-factor model demonstrated marginally acceptable fit to the data: χ^2^(524) = 4295.84, CFI = 0.907, TLI = 0.894, RMSEA = 0.051 (90% CI [0.050, 0.052]), SRMR = 0.052. Composite reliability (CR) values ranged from 0.64 (IC) to 0.90 (PES), and average variance extracted (AVE) values ranged from 0.37 (IC) to 0.70 (PES).

The Chinese version of the Maslach Burnout Inventory-Human Services Survey (MBI-HSS) includes three facets: emotional exhaustion (EE, 9 items), depersonalization (DP, 5 items), and reduced personal accomplishment (RPA, 8 items, with reversed scoring) (Maslach and Jackson, 1981). Each item’s responses are rated on a 6-point Likert scale from 1 (never) to 6 (every day). Higher scores indicate increased levels of occupational burnout, with higher scores reflecting more severe burnout. Cronbach’s alphas for the three subscales were 0.85 (EE), 0.77 (DP), and 0.87 (RPA). CFA demonstrated acceptable fit to the data: χ^2^(206) = 4379.64, CFI = 0.856, TLI = 0.839, RMSEA = 0.086 (90% CI [0.083, 0.088]), SRMR = 0.087. CR values ranged from 0.78 (DP) to 0.86 (RPA), and AVE values ranged from 0.45 (DP) to 0.47 (RPA).

### 2.4 Statistical analysis

Mplus 8.3, SPSS 26.0, and R software (version 4.3.1) were used to analyze the data. Descriptive statistics for all the main variables were computed, with teacher stress and occupational burnout expressed as means and standard deviations (SD), and gender, age, educational background, and job tenure computed as frequencies and percentages.

To identify distinct subgroups of teacher stress and occupational burnout, Latent Profile Analysis (LPA) was employed based on underlying latent factors. Model fit was appraised through various criteria, including the Akaike Information Criterion (AIC), Bayesian Information Criterion (BIC), and adjusted Bayesian Information Criterion (aBIC). Lower values on the aforementioned indices indicate a better-fitting model. Classification accuracy was assessed using Information Entropy, with values close to 1 preferred. The Bootstrapped Likelihood Ratio Test (BLRT) and Lo-Mendell-Rubin Likelihood Ratio Test (LMRT) facilitated comparisons between models with differing numbers of profiles, where significant *p*-values suggested an enhanced fit for the k-class model (Vermunt, 2024).

Network analysis was performed to visualize the interrelationships among the latent profiles, utilizing the R package bootnet (Hevey, 2018). A Gaussian graphical model was utilized to estimate networks (Epskamp et al., 2018). In these distinct networks, nodes represented different latent variables of stress and burnout, while edges indicated the conditional correlations between them. The Least Absolute Shrinkage and Selection Operator (LASSO) and the Extended Bayesian Information Criteria (EBIC) were used to simplify the network by reducing unnecessary edges and selecting relevant tuning parameters (Zhou D. J. et al., 2024). Thicker edges showed stronger connections between nodes. To formally compare network structures between profiles, a Network Comparison Test (NCT) was conducted, examining network structure invariance, global strength, individual edge weights, and centrality indices (Van Borkulo et al., 2023), with 1000 permutation iterations and false discovery rate (FDR) correction. Edge accuracy was assessed via nonparametric bootstrapping (1,000 samples), and 95% confidence intervals were computed for edge weights. The stability of centrality measures was evaluated utilizing a case-dropping bootstrap procedure (1,000 samples), with an expectation for the correlation stability (CS) coefficient to exceed 0.25, and values surpassing 0.5 deemed optimal (Epskamp et al., 2018).

## 3 Results

### 3.1 Sample description

Descriptive demographic information is provided in Table 1. Overall, the majority of subjects were female (66.9%). A significant proportion (83.7%) held a bachelor’s degree. The sample was fairly evenly distributed across the four age groups and four ranges of occupational tenure.

### 3.2 Descriptive and correlation analysis

Table 2 presents the correlation matrix for the main variables, including gender, age, occupational tenure, education level, three facets of occupational burnout, and the nine categories of teacher stress. The three facets of occupational burnout and the nine classes of teacher stress were all significantly positively correlated, indicating strong relationships between the nine classes of stressors and the three facets of burnout.

**TABLE 2:** Correlations of variables.

Variable	1	2	3	4	5	6	7	8	9	10	11	12	13	14	15
1. Gender	–	–	–	–	–	–	–	–	–	–	–	–	–	–	–
2. Age	−0.246***														
3. Tenure	−0.231***	0.865***													
4. Edu	0.148***	−0.640***	−0.713***												
5. EE	−0.054**	0.090***	0.119***	−0.050**											
6. DP	−0.105***	0.058**	0.078***	0.019	0.633***										
7. RPA	−0.055**	0.074***	0.091***	−0.069***	0.177***	0.267***									
8. SB	−0.040	0.003	0.007	0.034	0.365***	0.307***	0.293***								
9. EAR	−0.130***	0.048*	0.070***	−0.016	0.421***	0.448***	0.309***	0.412***							
10. TTR	−0.105***	0.078***	0.098***	−0.024	0.423***	0.467***	0.355***	0.472***	0.709***						
11. PTR	−0.033	0.000	0.005	0.013	0.391***	0.333***	0.255***	0.527***	0.452***	0.554***					
12. TM	−0.007	−0.002	0.020	0.032	0.498***	0.378***	0.300***	0.512***	0.516***	0.533***	0.576***				
13. IC	0.035	−0.007	0.021	0.016	0.405***	0.272***	0.212***	0.524***	0.433***	0.477***	0.542***	0.638***			
14. PS	0.004	0.110***	0.151***	−0.097***	0.616***	0.388***	0.303***	0.445***	0.426***	0.438***	0.484***	0.622***	0.596***		
15. PES	0.025	−0.006	0.031	0.003	0.587***	0.494***	0.387***	0.520***	0.537***	0.570***	0.545***	0.628***	0.601***	0.726***	
16. SM	0.000	0.054**	0.084***	−0.075***	0.360***	0.309***	0.349***	0.187***	0.298***	0.333***	0.254***	0.322***	0.201***	0.344***	0.440***

Edu, education level; EE, emotional exhaustion; DP, depersonalization; RPA, reduced personal achievement; SB, Student Behavior; EAR, Employee and Administrator Relationships; TTR, teacher-and-teacher relations; PTR, Parent and Teacher Relationships; TM, time management; IC, Intrapersonal Conflicts; PS, physical symptoms of stress; PES, psychological or emotional symptoms of stress; and SM, Stress Management Techniques. The same applies hereinafter. * indicates *p* < 0.05, ** indicates *p* < 0.01, *** indicates *p* < 0.001.

### 3.3 Latent profiles analysis of burnout and stress

To explore the latent profiles of stress and occupational burnout, the mean score of the three facets of occupational burnout and nine classes of stress were used for the LPA, and the latent profile models for one to four profiles were established. The fit indices of the models are shown in Table 3. The entropy values for all models were greater than 0.85, with the highest entropy value of 0.895 observed in the two-profile model. The AIC, BIC, and aBIC showed a continuous decrease as the number of profiles increased. Both the LMR-LRT and the BLRT were significant for the two-profile and three-profile models. Based on the principle of model parsimony (Marsh et al., 2020), the two-profile model was identified as the best-fitting model.

**TABLE 3:** Model fit indices for latent profile analysis.

Model	AIC	BIC	aBIC	Entropy	LMRLRT	BLRT	Class proportion (%)
1	146879.62	147021.84	146945.59				1
2	137166.88	137386.14	137268.58	0.895	< 0.001	< 0.001	64.34/35.66
3	133749.94	134046.23	133887.37	0.886	< 0.001	< 0.001	37.61/46.46/15.93
4	132684.75	133058.08	132857.91	0.856	0.085	< 0.001	41.11/28.59/22.30/8.00

**FIGURE 1:**
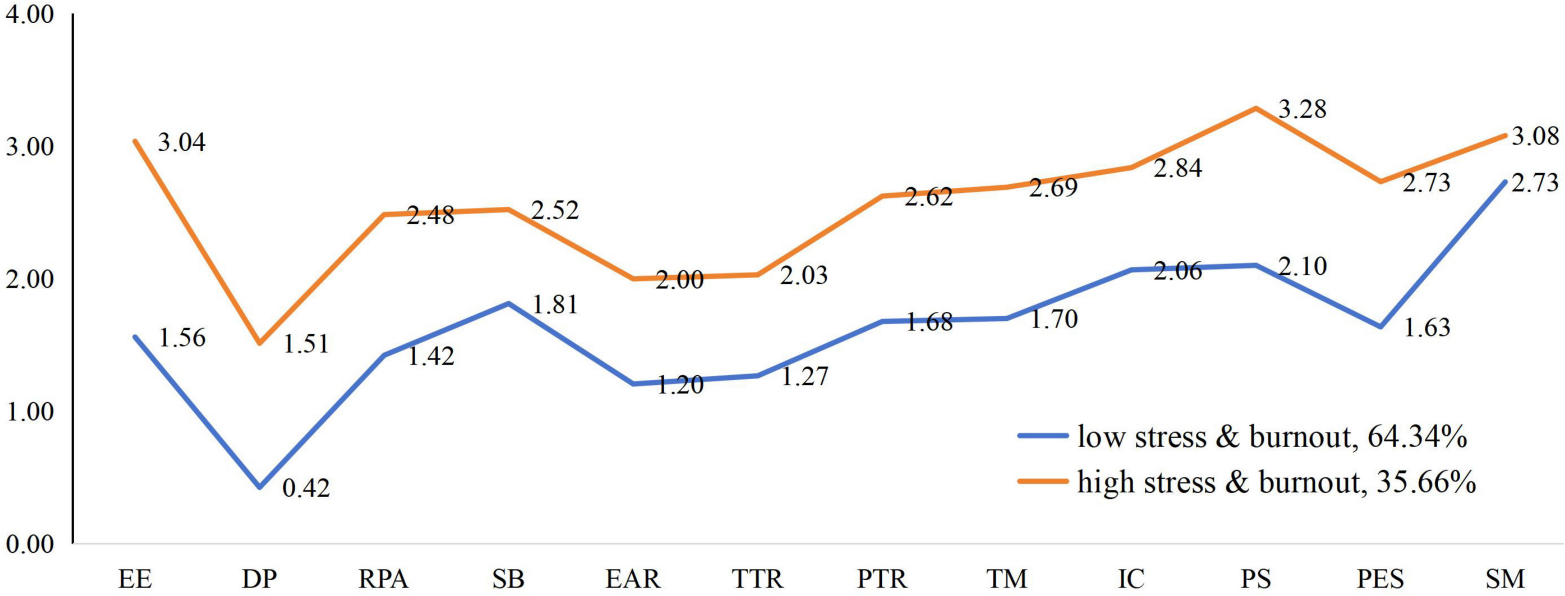
Estimated means of the teacher stress and occupational burnout in two profiles identified through latent profile analysis. The *x*-axis represents the latent factors of occupational burnout and teacher stress, while the *y*-axis represents the mean score.

The two-profile model was selected based on goodness-of-fit indices and substantive interpretability. Examination of the profile means indicated that the two groups differed primarily in the overall severity of stress and occupational burnout. The larger subgroup (64.34%) showed relatively low scores across all stress and burnout dimensions and was therefore labeled the low stress-burnout subgroup. In contrast, the smaller subgroup (35.66%) showed consistently higher scores across all dimensions and was labeled the high stress-burnout subgroup (see Figure 1).

### 3.4 Network analysis for two-profile

**FIGURE 2:**
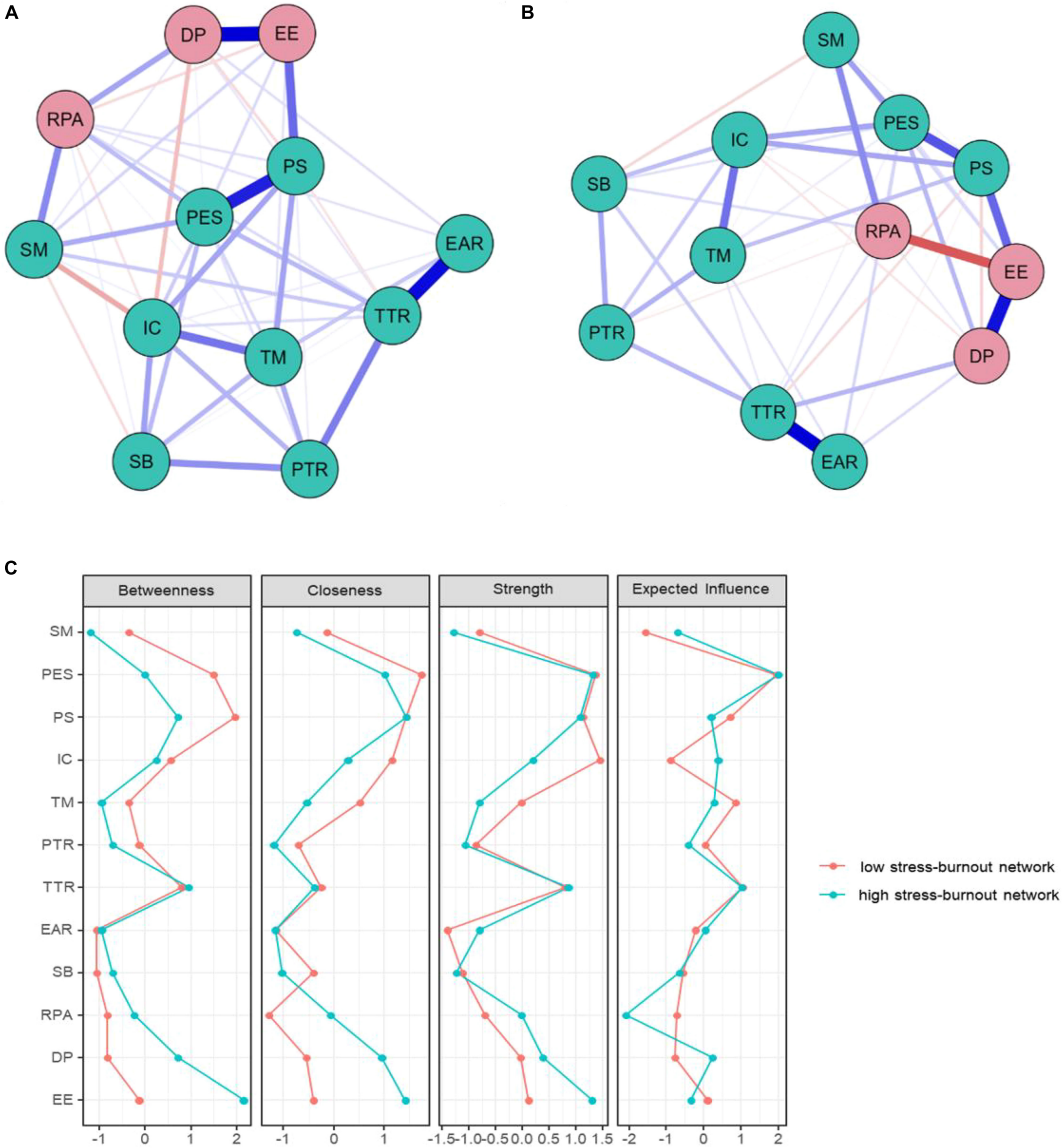
Networks of teacher stress and occupational burnout. An undirected network of teacher stress and occupational burnout among low levels of teacher stress and burnout **(A)** and high levels of teacher stress and burnout **(B).** Blue lines indicate positive correlations and red lines indicate negative correlations for panels **(A,B)**. Thicker edges represent stronger associations. **(C)** Standardized centrality indices for each node in the low stress-burnout and high stress-burnout networks.

Figures 2A, B illustrate the unadjusted network for the stress-burnout profiles of the low and high co-occurrence subgroups.

Of 66 overall possible edges, in the low stress-burnout network 54 edges were significant; while in the high stress-burnout network, 48 edges were significant. In the low stress-burnout network, the strongest edge was identified between EE and DP (0.420), TTR and EAR (0.404), PES and PS (0.371). In the high stress-burnout network, the most robust edge was observed between TTR and EAR (0.476), DP and EE (0.444), RPA and EE (−0.316). The weighted adjacency matrix is shown in Supplementary Table 1. The stability and robustness of the stress-burnout networks in the low and high stress-burnout subgroups were examined (see Supplementary Figure 1).

In the low stress-burnout network, one node with the highest betweenness was PS (*r*_*s*_ = 1.96), the highest closeness was PES (*r*_*s*_ = 1.75), and the highest strength was IC (*r_*s*_* = 1.46). In the high stress-burnout network, the one node with the highest betweenness was EE (*r*_*s*_ = 2.15), with the highest closeness was PS *(r_*s*_* = 1.45), and with the highest strength was PES (*r*_*s*_ = 1.31). In both low and high stress-burnout networks, the node with the highest expected influence (EI) index was PES (low stress-burnout network, *r_*s*_* = 1.96; high stress-burnout network, *r*_*s*_ = 2.01). In the low-stress network, the node with the lowest EI value was SM (*r*_*s*_ = −1.57), whereas in the high-stress network, the node with the lowest EI value was RPA (*r*_*s*_ = −2.09). The stress-burnout network of the low stress-burnout and high stress-burnout subgroups investigated robust stability (see Supplementary Figure 2).

To formally test profile differences, a Network Comparison Test (NCT) was conducted with 1000 permutation iterations and FDR correction. The network structure invariance test indicated that the two networks differed significantly in overall topology (M = 0.28, *p* = 0.002). However, the global strength invariance test was not significant (S = 0.77, *p* = 0.108), suggesting comparable overall connectivity. Edge-wise comparisons revealed no significant differences after FDR correction, though the edges between EE-RPA, DP-RPA, and IC-SM did not survive FDR correction but exhibited marginally significant trends (uncorrected *p* < 0.04). Centrality invariance tests further indicated that several nodes differed significantly between groups, including emotional exhaustion, time management, and stress management in strength and expected influence (*p* < 0.011).

Centrality analysis revealed that in the low stress-burnout network, PHC showed the highest betweenness (1.96), PES the highest closeness (1.75), and IC the highest strength (1.46). In the high stress-burnout network, EE showed the highest betweenness (2.15), PHC the highest closeness (1.45), and PES the highest strength (1.31). In both networks, PES demonstrated the strongest expected influence (1.96 and 2.01, respectively), whereas SM and RPA showed the lowest expected influence in the low (−1.57) and high (−2.09) stress-burnout networks, respectively.

Network stability was assessed via case-dropping bootstrapping (1,000 samples). Correlation stability (CS) coefficients for the low stress-burnout network were 0.52 (betweenness), 0.59 (closeness), 0.75 (expected influence), and 0.75 (strength); for the high stress-burnout network, CS coefficients were 0.59 (betweenness), 0.67 (closeness), 0.75 (expected influence), and 0.75 (strength). All values exceeded the recommended threshold of 0.25 and approached or exceeded the optimal threshold of 0.50, indicating robust stability. Edge accuracy was evaluated via nonparametric bootstrapping (1,000 samples); the 95% confidence intervals for key edges excluded zero (e.g., EE–DP: [0.240, 0.326] in the low group and [0.307, 0.440] in the high group; RPA–EE: [-0.355, -0.217] in the high group), confirming satisfactory estimation precision.

## 4 Discussion

Teachers were classified into two severity-based subgroups according to their levels of stress and occupational burnout: a low stress-burnout subgroup and a high stress-burnout subgroup. Although the two groups primarily differed in the overall severity of stress and burnout, network analysis revealed some differences in the patterns of associations among stress and burnout dimensions. These findings contribute to understanding how stress and occupational burnout are associated across different levels of severity and may provide preliminary insights for future intervention research.

### 4.1 Stress-burnout subgroup characteristics

This study examined whether Chinese teachers could be classified into subgroups with different levels of stress and occupational burnout using LPA. The LPA results identified two subgroups that differed primarily in the severity of stress and occupational burnout: the “low stress-burnout” subgroup and the “high stress-burnout” subgroup. The low stress-burnout subgroup exhibited relatively low levels of both teacher stress and occupational burnout symptoms. In contrast, the high stress-burnout subgroup reported significantly higher levels of both stress and occupational burnout, with elevated scores across all latent dimensions compared to the low stress-burnout subgroup. This finding suggests that teachers differ substantially in the severity of stress and occupational burnout symptoms, indicating that a substantial proportion of teachers experience elevated levels of stress and occupational burnout and may therefore be at greater risk for impaired well-being.

More than one-third of the teachers in this study reported high levels of both occupational stress and occupational burnout, which is consistent with previous studies conducted among teachers in different educational contexts (Bermejo-Toro and Prieto-Ursúa, 2014; Herman et al., 2018; Zhong et al., 2009). These findings highlight the importance of continued attention to teachers’ occupational well-being and suggest that teachers experiencing elevated levels of stress and burnout may benefit from additional organizational and psychological support.

Although the two subgroups differed primarily in overall severity, emotional exhaustion (EE) and physical symptoms of stress (PS) showed particularly high levels in both subgroups. This finding is significant for several reasons. On the one hand, emotional exhaustion, a main facet of burnout, is often regarded as the most direct and substantial indicator of occupational burnout (Sriwilai and Charoensukmongkol, 2016; Zhao and Jiang, 2022). It represents the depletion of emotional resources due to excessive work demands, leading to fatigue, reduced energy, and diminished motivation (Maslach and Jackson, 1981; Maslach and Leiter, 2016; Park and Shin, 2020). Meanwhile, given that emotional exhaustion significantly influences teachers’ attitudes and behaviors, this dimension may warrant particular attention in future research and prevention efforts (Zhao and Jiang, 2022; Innstrand et al., 2004). Research focused on emotional exhaustion is crucial for understanding the psychological toll of teaching and for developing targeted interventions that can alleviate burnout symptoms.

On the other hand, the presence of physical symptoms of stress - such as psychogenic headaches, chronic digestive issues, loss of appetite, or low arousal (Burke et al., 2020) - reflects the physiological impact of stress among teachers (Austin et al., 2005; Luh et al., 1991). These symptoms are particularly prevalent among teachers, given the sedentary nature of their work, which often involves prolonged periods of sitting while engaged in activities like lesson planning and grading (Perlito et al., 2021). Such activities, although mentally taxing, can also lead to physical stress responses (Bakhtari Aghdam et al., 2016; Bermejo-Toro and Prieto-Ursúa, 2014; Wei et al., 2024). These findings highlight that stress and burnout involve both psychological and physiological manifestations and should therefore be considered from a multidimensional perspective.

### 4.2 Network analysis of stress and burnout

NA examined the co-occurrence of stress and occupational burnout symptoms in teachers. The results indicated that the network structures of the two stress-burnout subgroups were broadly similar. Nodes within these networks showed strong conditional associations, reflecting a pattern of conditional dependence among symptoms. This high level of connectivity indicates that stress and occupational burnout symptoms are statistically interrelated and tend to form clusters of associated features within the network structure (Bermejo-Toro and Prieto-Ursúa, 2014; Bereznowski et al., 2023).

Centrality analysis identified the most central nodes within the networks. In both the low and high stress-burnout networks, psychological and emotional symptoms of stress (PES) and teacher-and-teacher relations (TTR) were identified as the most influential nodes, with the highest expected influence (EI) values. These nodes showed high centrality within the networks, as reflected by their EI scores. Psychological and emotional symptoms of stress, such as anxiety, frustration, and emotional exhaustion, have been recognized for their relevance to teachers’ mental and physical health (Maddock, 2024; Suyi et al., 2017; Zhao and Jiang, 2022; Zhong et al., 2009). Within the present networks, these symptoms showed strong connections with other stress and burnout-related variables (Guthier et al., 2020).

It should be noted that teacher-and-teacher relations (TTR) showed relatively high centrality in both networks, indicating that this node is strongly connected within the network structure. Previous research on teacher-and-teacher relations in burnout remains limited, although it is increasingly considered an important contextual factor in occupational stress research. Poor interpersonal relationships among colleagues, together with competition and limited resources within schools, are often associated with higher levels of stress and burnout (Mohammed et al., 2023). Issues related to faculty evaluation, performance assessments, and limited collaborative practices may be associated with less efficient work patterns and higher perceived stress. Teachers are often required to perform tasks independently without adequate support from colleagues (Teng et al., 2024), which may be associated with greater feelings of isolation and stress (Zhong et al., 2009). Positive interpersonal relationships and collaborative communication among teachers have been associated with lower levels of work-related stress (Schwartz and Minkov, 2023). Schools that promote collaboration and supportive communication may also be associated with lower levels of teacher burnout, suggesting that interpersonal dynamics represent a relevant contextual factor in teacher well-being.

A noteworthy finding from the network analysis was the robust positive association between emotional exhaustion (EE) and depersonalization (DP), which emerged as one of the strongest edges in both networks. Emotional exhaustion reflects the depletion of emotional resources due to excessive work demands, whereas depersonalization refers to a detached and impersonal attitude toward work-related interactions (Maslach and Jackson, 1981; Zhao and Jiang, 2022). The stability of this positive edge across both profiles underscores the intrinsic, synergistic coupling between the emotional and interpersonal dimensions of burnout. Concretely, in the educational context, this robust partial correlation indicates that a teacher’s emotional exhaustion and their psychological detachment from students, colleagues, and professional roles are mutually reinforcing components within the same symptom network, rather than isolated sequential events.

In addition, a negative association was identified between reduced personal accomplishment (RPA) and emotional exhaustion (EE) in the high stress-burnout subgroup. RPA reflects a diminished sense of competence and achievement in one’s professional role (Maslach and Jackson, 1981), whereas EE reflects the depletion of emotional resources due to chronic work demands. This finding suggests that the associations among burnout dimensions may not be uniform under conditions of elevated stress and burnout, highlighting the multidimensional nature of burnout.

Furthermore, the formal Network Comparison Test (NCT) revealed a significant divergence in overall network topology between the low and high stress-burnout subgroups, despite a comparable global strength. This distinct pattern implies that while the overall density of symptom co-occurrence remains uniform across different severity levels, the structural architecture and clustering profile within the symptom network undergo a qualitative shift as burnout intensifies. These findings underscore that the transition from low to high stress-burnout profiles is characterized not merely by a linear escalation in symptom severity, but more fundamentally by a structural reorganization of symptom interrelationships, providing empirical justification for tailoring profile-specific intervention strategies.

### 4.3 Implication for practice

This study has practical implications. Firstly, the results highlight the significance of addressing the multiple stressors faced among teachers in China, particularly stress related to students’ home environments. School administrators and policymakers should consider strategies to alleviate this type of stress, such as increasing collaboration between schools and families, implementing support programs for students from disadvantaged backgrounds, and providing teachers with resources to navigate challenging home situations (Ali et al., 2024; Beausaert et al., 2016). By reducing external stressors related to students’ home environments, schools were suggested to build a more friendly working environment for teachers (Bereznowski et al., 2023). Secondly, the network analysis results emphasize the critical role of emotional exhaustion and depersonalization in teacher burnout. School leadership should prioritize programs that provide emotional support for teachers, such as stress-reliever programs focused on stress management and emotional well-being. Additionally, fostering a culture of appreciation and recognition within schools can help boost teachers’ sense of accomplishment and reduce feelings of burnout. These efforts can improve teachers’ well-being, enhance professional satisfaction, and reduce turnover rates (Gu et al., 2015).

### 4.4 Limitations and future research direction

At least three limitations should be acknowledged. First, the sample from a single source may limit the generalizability. The data were collected from a specific province in China, which may not fully represent the diversity of educational settings across different regions. Future studies should include more diverse samples, such as teachers from various countries, school types (e.g., public vs. private), and socio-economic backgrounds, to enhance the robustness and generalizability. Second, the cross-sectional, self-report data preclude inferences regarding causality or directionality. Network edges and centrality indices estimated within the GGM framework represent concurrent partial correlations—providing a snapshot at a single point in time—rather than dynamic processes of symptom activation. Consequently, highly central or bridge nodes should not be misinterpreted as proven causal drivers or definitive intervention targets. Instead, they offer exploratory candidate clues for hypothesis generation, which require further validation through future longitudinal or experimental designs. Finally, while LPA combined with network analysis offers a sophisticated approach to exploring the interconnections between latent variables, it does not account for potential confounding factors that could affect the relationships between teacher stress and occupational burnout. Future studies could incorporate additional variables, such as work-life balance, personal resilience, and school leadership support, to find the factors contributing to stress and occupational burnout among teachers.

## 5 Conclusion

In conclusion, two subgroups characterized by different levels of stress and occupational burnout were identified among Chinese teachers. Rather than representing qualitatively distinct profiles, these subgroups primarily differed in the overall severity of stress and burnout symptoms. Network analysis indicated that the two severity-based subgroups shared comparable overall connectivity but differed in several specific associations, node characteristics, and overall network topology. These findings contribute to a better understanding of the co-occurrence of teacher stress and occupational burnout and provide preliminary evidence regarding potentially influential symptoms that may warrant further attention in future research. The results may also inform school administrators, policymakers, and mental health professionals when designing support programs aimed at promoting teacher well-being and reducing occupational burnout.

## Data Availability

The raw data supporting the conclusions of this article will be made available by the authors, without undue reservation.
