# Supplementary material for: Occupational stress and burnout among teachers in China: latent profile and network analyses
Source: Front Psychol. 2026 Jul 29;17:1862159. doi: 10.3389/fpsyg.2026.1862159 (PMC13461310; doi:10.3389/fpsyg.2026.1862159)
Supplement: Supplementary file 1 [file Supplementary_file_1.docx]

**Supplementary Material**

**TABLE S1 |** The full weighted adjacency matrix for low stress-burnout network (left) and high stress-burnout network (right).

| Variable | EE | DP | RPA | SB | EAR | TTR | PTR | TM | IC | PS | PES | SM |
| --- | --- | --- | --- | --- | --- | --- | --- | --- | --- | --- | --- | --- |
| EE | \ | 0.444 | -0.316 | 0.000 | 0.000 | -0.012 | 0.000 | 0.007 | 0.000 | 0.287 | 0.063 | 0.081 |
| DP | 0.423 | \ | 0.047 | 0.014 | 0.073 | 0.142 | 0.000 | 0.000 | -0.056 | -0.086 | 0.139 | 0.000 |
| RPA | -0.066 | 0.155 | \ | 0.074 | 0.000 | 0.066 | -0.046 | 0.000 | -0.043 | -0.018 | 0.079 | 0.221 |
| SB | 0.000 | 0.000 | 0.034 | \ | 0.000 | 0.102 | 0.168 | 0.000 | 0.129 | 0.000 | 0.063 | -0.081 |
| EAR | 0.000 | 0.058 | 0.053 | 0.028 | \ | 0.476 | 0.000 | 0.063 | 0.000 | -0.032 | 0.083 | 0.000 |
| TTR | 0.000 | 0.046 | 0.065 | 0.020 | 0.404 | \ | 0.141 | 0.051 | 0.012 | -0.072 | 0.034 | 0.000 |
| PTR | -0.011 | -0.007 | 0.000 | 0.188 | 0.000 | 0.217 | \ | 0.152 | 0.106 | -0.018 | 0.036 | 0.000 |
| TM | 0.051 | 0.000 | 0.023 | 0.126 | 0.101 | 0.000 | 0.142 | \ | 0.27 | 0.123 | 0.04 | 0.024 |
| IC | -0.021 | -0.112 | -0.054 | 0.161 | 0.036 | 0.066 | 0.129 | 0.232 | \ | 0.171 | 0.172 | 0.000 |
| PS | 0.251 | -0.066 | 0.055 | 0.000 | 0.000 | -0.053 | 0.037 | 0.158 | 0.154 | \ | 0.323 | 0.03 |
| PES | 0.067 | 0.026 | 0.115 | 0.122 | 0.004 | 0.121 | 0.056 | 0.073 | 0.092 | 0.371 | \ | 0.181 |
| SM | 0.073 | 0.044 | 0.198 | -0.064 | 0.007 | 0.095 | 0.000 | 0.034 | -0.144 | 0.000 | 0.142 | \ |

a. low stress-burnout network

b. high stress-burnout network

**Figure S1 |** Bootstrapped confidence intervals of edge weights.

Note. The black dots represent the edge weight values, which are arranged from highest to lowest. The gray region shows the edge weights’95% Confidence Intervals. Wide intervals indicate less stability, whereas tight intervals denote more stability.

a. low stress-burnout network

b. high stress-burnout network

**Figure S2 |** The centrality stability of network structure by case dropping bootstrap.

Note. The dots represent the edge weight values, which are arranged from highest to lowest. The gray region shows the edge weights’ 95% Confidence Intervals. Wide intervals indicate less stability, whereas tight intervals denote more stability.
